# Supplementary material for: ROS-Mediated Necroptosis Promotes Coxsackievirus B3 Replication and Myocardial Injury
Source: Microorganisms. 2025 Oct 17;13(10):2389. doi: 10.3390/microorganisms13102389 (PMC12566541; doi:10.3390/microorganisms13102389)
Supplement: Supplementary file 1 [file microorganisms-13-02389-s001.zip › microorganisms-3797893-Supplementary Table 1.docx]

Table S1 The antibodies used in the present study

| Antibody | Dilution ratio | Resource |
| --- | --- | --- |
| RIP1 | 1:1000 | Beyotime, China |
| RIP3 | 1:1000 | Abcam, USA |
| p-MLKL | 1:2000 | Abcam, USA |
| MLKL | 1:1000 | Proteintech, China |
| GAPDH | 1:2000 | Proteintech, China |
| Nrf2 | 1:1000 | Beyotime, China |
| HO-1 | 1:2000 | Beyotime, China |
