# Supplementary material for: ROS-Mediated Necroptosis Promotes Coxsackievirus B3 Replication and Myocardial Injury
Source: Microorganisms. 2025 Oct 17;13(10):2389. doi: 10.3390/microorganisms13102389 (PMC12566541; doi:10.3390/microorganisms13102389)
Supplement: Supplementary file 1 [file microorganisms-13-02389-s001.zip › microorganisms-3797893-Supplementary Table 2.docx]

Table S2 Gene primers used in RT-qPCR

| Gene | Forward Primer | Reverse Primer |
| --- | --- | --- |
| CVB3 | GCACACACCCTCAAACCAGA | ATGAAACACGGACACCCAAAG |
| IL-1β (Mouse) | AAAGACCTCTATGCCAACACAGT | CTGACTTGGCAGAGGACAAAG |
| IL-6 (Mouse) | TAGTCCTTCCTACCCCAATTTCC | TTGGTCCTTAGCCACTACTTC |
| TNF-α (Mouse) | TCAAGTGGCATAGATGTGGAAGAA | TGGCTCTGCAGGATTTTCATG |
| IFN-α (Mouse) | GTCACTACGAATCGCACCTGATCACT | CCGATGTATAGACATTCCTCTGG |
| GAPDH (Mouse) | AGGGCATCTTGGGCTACAC | CATACCAGGAAATGAGCTTGA |
| GAPDH (Human) | GCACCGTCAAGGCTGAGAAC | TGGTGAAGACGCCAGTGGA |
